# Factors associated with the use of dietary supplements and over-the-counter medications in Japanese elderly patients

**DOI:** 10.1186/s12875-017-0699-9

**Published:** 2018-01-24

**Authors:** Shoichi Masumoto, Mikiya Sato, Takami Maeno, Yumiko Ichinohe, Tetsuhiro Maeno

**Affiliations:** 1 Department of Primary Care and Medical Education, Graduate School of Comprehensive Human Sciences, University of Tsukuba, 1-1-1 Tennodai, Tsukuba, Ibaraki 305-8577 Japan; 2 Kawakita Center for Family Medicine, Kawakita General Hospital, 1-7-3, Asagayakita, Sughinamiku, Tokyo, 166-0001 Japan; 3 Department of Health Services Research, Faculty of Medicine, University of Tsukuba, 1-1-1 Tennodai, Tsukuba, Ibaraki 305-8577 Japan

**Keywords:** Dietary supplements, Over-the-counter drugs, Nonprescription medications, Elderly, Polypharmacy

## Abstract

**Background:**

The use of dietary supplements and over-the-counter (OTC) drugs is increasing, and there is adequate concern about potential harmful effects. However, there are limited reports on the concurrent use of nonprescription medications with prescription medications in elderly patients. Therefore, this study was conducted to describe the use of dietary supplements and OTC drugs, and to identify predictors for their use in elderly patients using medications prescribed for chronic diseases.

**Methods:**

This was a cross-sectional study that enrolled 729 patients aged ≥65 years with chronic diseases, between January and March 2016. Data regarding socio-demographic status, medical condition, number of prescriptions, use of nonprescription medications, and psychological status were collected using a self-administered questionnaire and by review of medical records. Data regarding use of dietary supplements and OTC drugs were analyzed using descriptive statistics. Logistic regression analysis was applied to investigate factors associated with the use of dietary supplements and OTC drugs.

**Results:**

The regular use of nonprescription drugs was reported by 32.5% of patients. Vitamins were the most commonly used dietary supplements in elderly patients. Female sex, higher educational qualifications, and good economic status were identified as predictors for the use of nonprescription medications. Concurrent use of nonprescription medications with more than 5 prescription medications was detected in 12.2% of participants. The disclosure rate of the use of nonprescription medications by patients to the physician was 30.3%.

**Conclusion:**

The use of dietary supplements and OTC drugs was common in elderly patients with chronic diseases, and its use is associated with sex, education, and economic status. General practitioners (GPs) need to recognize the potential use of nonprescription medications, considering that polypharmacy was common and disclosure rate was low in this study.

**Electronic supplementary material:**

The online version of this article (10.1186/s12875-017-0699-9) contains supplementary material, which is available to authorized users.

## Background

Recent studies have shown that the prevalence of over-the-counter (OTC) medications and use of dietary supplements in older adults is increasing [1, 2]. The use of such drugs and products by elderly patients with chronic diseases needs to be particularly considered, because the drug metabolism in these patients is deteriorated due to their age. Nevertheless, concurrent use of nonprescription medications with prescription medications is common according to a study conducted in the United States (U.S.) [3]. It is difficult for medical practitioners to know the use of nonprescription medications as these are used at the discretion of the patients. In addition, previous reports suggested that many patients taking nonprescription medication do not tend to disclose the use of these drugs to their physician [4, 5].

A systematic review about self-medication in the elderly population reported that the prevalence of self-medication varied between 4 and 87%, and was associated with female sex, visits to pharmacists, depression, functional dependency, recent hospitalization, oral pain, restriction of activities and physical inactivity [6]. Another research study conducted in the U.S. reported that factors associated with dietary supplement use in patients taking prescription medication were female sex, Hispanic ethnicity, higher education, lack of medical insurance, and chronic conditions [7]. While depression was associated with the use of complementary medications in cancer patients [8], only one study reported the association of depression with the use of multiple nonprescription medications in the elderly population [9]. Overall, the association of anxiety or depression with the use of nonprescription medications was not well assessed in previous studies in elderly patients with chronic diseases.

Most of the previous studies on the use of nonprescription medications were conducted in a limited amount of countries and with areas mainly in the U.S. or Brazil, and reports from Asian countries are few. The use of nonprescription medications can be different per country and area, as it is dependent on the access to medical care, health system, and cultural differences. However, the use of dietary supplements and OTC medications and the predictors for its use are not well studied in Japanese elderly patients.

In this study, we aimed to describe the use of nonprescription medications including dietary supplements and OTC medications, and additionally identify the predictors for its use in elderly patients who use prescription medications for chronic diseases in Japan. We also hypothesized that depression or anxiety is associated with the use of nonprescription medications.

## Methods

### Study design and setting

This was a cross-sectional study conducted in an outpatient clinic at the Family Medicine department of an urban general hospital in Tokyo, Japan. Patients aged ≥65 years with chronic diseases who had at least one prescription medication were recruited between January and March 2016. Patients who met the following criteria were included in the study: age ≥ 65 years, presence of chronic diseases, and use of at least one prescription medication (excluding topical drugs). Written informed consent was obtained from each participant and those who did not consent to the study were excluded. Patients younger than 65 years, and patients with difficulty in communicating in Japanese were excluded.

### Data collection

Data from electronic medical records and responses to the questionnaire were used to obtain information about the patients’ characteristics including age, sex, estimated glomerular filtration rate (mL/min/1.73 m^2^), subjective economic status, educational qualifications, Hospital Anxiety and Depression Scale (HADS) score, use of nonprescription medications, and disclosure of the use to a physician. According to the common definition [10], polypharmacy in our study was defined as prescription of more than 5 drugs. Comorbidity was assessed using the Charlson Comorbidity Index (CCI), with the information obtained from the patients’ medical records and the questionnaire survey. The CCI was developed in 1987 and has been used since as a tool to assess the severity of chronic diseases [11]. The HADS was used to study the relationship between psychological status and the use of nonprescription medications. The HADS was developed by Zigmond and has been used worldwide to measure anxiety and depression in patients with chronic diseases [12]. It consists of 14 items divided between 2 question groups, one on anxiety, and one on depression, with each question rated from 0 to 3. A Japanese version was validated in a previous study [13]. We used a score of 8 points as a cut-off for both evaluation of anxiety and depression, i.e., patients with a score of 8 points or more on the anxiety or depression scales, were identified as anxious and depressed, respectively.

### Outcome measurements

Questions about current use of nonprescription medications were part of the questionnaire (“Currently, do you regularly use OTCs, dietary supplements, healthy foods, or herbal medicine other than prescriptions by doctors?”), Of those who answered yes, respondents were asked the type of nonprescription medications, divided into 4 categories, i.e., OTC medications, dietary supplements including vitamins and healthy foods, herbal medicines and others. There was no definition for dietary supplements in Japan, therefore we defined them as “A dietary supplement is a product intended for ingestion that contains a "dietary ingredient" intended to add further nutritional value to (supplement) the diet.” using definition by U.S. Food and Drug Administration (FDA) [14]. Patients who use nonprescription medications regularly were asked whether they had disclosed the use to their physician (“Have you told to your doctor that you have been using OTCs, dietary supplements, healthy foods, or herbal medicine other than prescriptions by doctors?”.

### Statistical analysis

Descriptive data are presented by mean and standard deviation (SD) for normally distributed continuous variables, and by median and interquartile range (IQR) for non-parametric variables. Categorical data are presented as number and percentage. The percentage of categorical variables were compared using χ^2^ tests. Variables with a *P* value below 0.10, anxiety and depression were used for logistic regression analysis to assess the association between each independent variable and the use of nonprescription medications. The results of the logistic regression analysis are presented with odds ratios (ORs) and 95% confidence intervals (CIs). A *P* value below 0.05 was considered to be statistically significant. All statistical analyses were performed using IBM SPSS ver. 22 (IBM Corp., Armonk, NY, USA).

## Results

### Background data of patients

A total of 729 patients were included in the analysis. Basic characteristics of participants are shown in Table 1. The mean age and ± SD of participants was 75.6 ± 7.5 years, and 51.6% of the population were women. The median number of prescription medications that was taken per patient was 4 (IQR 2–6) with a range of 1–22. Polypharmacy in patients in this study was 39.3%. The percentages of patients with a depressive mood and anxiety, who scored 8 points or more by the HADS, were 23.1% and 16.1%, respectively. Underlying medical conditions are shown in Table 2. The median CCI was 1 (IQR 0–1), which indicates that many participants had relatively mild diseases.

**Table 1:** Characteristics of the Study Population

Characteristics	Total (*n* = 729)
Age, mean ± SD	75.6 ± 7.5
Sex, n (%)	
Male	353 (48.4)
Female	376 (51.6)
Drug prescriptions per patient, median (IQR)	4 (2–6)
Charlson comorbidity index, median (IQR)	1 (0–1)
Smoking status, n (%)	
Current smoker	70 (9.6)
Past smoker	285 (39.3)
Never smoker	371 (51.1)
Regular drinker, n (%)	238 (32.8)
Lives alone, n (%)	187 (26.3)
Economic status, n (%)	
Less than average	122 (17.1)
Average	442 (62.0)
More than average	149 (20.9)
Educational qualification, n (%)	
≦High school	282 (40.3)
> High school	417 (59.7)
Anxiety by HADS, n (%)	106 (16.1)
Depression by HADS, n (%)	154 (23.1)

Missing values were omitted from percentage calculation

*SD* Standard deviation, *IQR* Interquartile range, *HADS* Hospital Anxiety and Depression Scale

**Table 2:** Underlying Medical Conditions of Patients

Underlying medical conditions	n (%)
Hypertension	525 (72.0)
Hyperlipidemia	374 (51.3)
Chronic Kidney Disease (eGFR < 50 mL/min/1.73m^2^)	153 (21.0)
Diabetes mellitus	150 (20.6)
Hyperuricemia/gout	138 (18.9)
Cerebrovascular disease	130 (17.8)
Respiratory disease (Bronchial Asthma, COPD)	86 (11.8)
Gastric ulcer	94 (12.9)
Dementia	57 (7.8)
Cardiovascular disease	51 (7.0)
Liver disease	39 (5.3)

*COPD* Chronic Obstructive Pulmonary Disease

### The use and details of nonprescription medications

Overall, 237 (32.5%) patients reported the use of nonprescription medications. Reported drugs and products were categorized into dietary supplements (28.0%), OTC medications (9.1%), and herbal medicines (3.6%). Vitamins and minerals, *aojiru* (green juice), and chondroitin-glucosamine were common in dietary supplements (Table 3). Of OTC drugs, digestives and laxatives were commonly used. The percentage of patients using herbal medicine was low. Only 72 (30.4%) patients disclosed the use of nonprescription medications out of 237 patients actually using them.

**Table 3:** Frequency of nonprescription medication use and details (*n* = 237)

Nonprescription medications	n (%)
Dietary supplements	204 (28.0)
Vitamin/minerals	48 (6.6)
Calcium	16 (2.2)
Vitamin C	12 (1.6)
Vitamin E	4 (0.5)
Any vitamin B	21 (2.9)
Chondroitin-glucosamine	29 (4.0)
Omega-3 fatty acids	9 (1.2)
Zinc	5 (0.7)
*Aojiru* (Green juice)	30 (4.1)
Coenzyme Q-10	3 (0.4)
Probiotics	8 (1.1)
Sesamin	12 (1.6)
Garlic	10 (1.4)
Hyaluronic acid/Collagen	15 (2.1)
Propolis/Royal Jerry	13 (1.8)
Vinegar	5 (0.7)
Blueberry, Carotenoids	13 (1.8)
Amino acids, Proteins	6 (0.8)
Combined natural products	24 (3.3)
Enzymes	9 (1.2)
Others	11 (1.5)
OTCs	66 (9.1)
Digestives	27 (3.7)
Laxative	14 (1.9)
Analgesics	5 (0.7)
Antihistamines	3 (0.4)
Herbal medicines	26 (3.6)
Not reported	39 (5.3)

### Predictors for the use of nonprescription medication

Univariate analysis showed that female sex, educational qualifications higher than high school, and good economic status were associated with the use of nonprescription medications. Anxiety and depression defined by the HADS score were not significantly associated with the use of nonprescription medications (Table 4). Female sex (Adjusted OR = 1.55, 95% CI = 1.01–2.37), educational qualifications higher than high school (Adjusted OR = 1.43, 95% CI = 1.14–2.44), and good economic status (Adjusted OR = 1.63, 95% CI = 1.08–2.46) were associated with the use of nonprescription medication on multivariable analysis, but neither anxiety nor depression were directly associated with the use of nonprescription medications (Table 5).

**Table 4:** Association between each variable and the use of nonprescription medications by univariate analysis

Variables	Nonprescription medication use		*P* value
	Yes: n, (%)	No: n, (%)	
Sex			0.013
Male	99 (28.0)	254 (72.0)	
Female	138 (36.7)	238 (63.3)	
Age			0.030
< 75	133 (36.2)	234 (63.8)	
≧75	104 (28.7)	258 (71.3)	
Number of prescription medications			0.454
≦4	148 (33.6)	293 (66.4)	
≧5	89 (30.9)	199 (69.1)	
CCI			0.312
0–1	184 (33.5)	365 (66.5)	
≧2	53 (29.4)	127 (70.6)	
Smoking status			0.017
Current smoker	16 (22.9)	54 (77.1)	
Past smoker	83 (29.1)	202 (70.9)	
Never smoker	138 (37.2)	233 (62.8)	
Drinking habit			0.951
Regular drinker	77 (32.4)	161 (67.6)	
Non drinker	159 (32.6)	329 (67.4)	
Family member			0.376
Lives alone	65 (34.8)	122 (65.2)	
Lives with other family members	164 (31.2)	361 (68.8)	
Economic status			0.002
Less than average	34 (27.9)	88 (72.1)	
Average	133 (30.1)	309 (69.9)	
More than average	67 (45.0)	82 (55.0)	
Educational qualification			0.003
≦High school	76 (27.0)	206 (73.0)	
> High school	157 (37.6)	260 (62.4)	
Anxiety by HADS			0.508
≦7	180 (32.5)	373 (67.5)	
≧8	38 (35.8)	68 (64.2)	
Depression by HADS			0.484
≦7	168 (32.7)	346 (67.3)	
≧8	55 (35.7)	99 (64.3)	

Missing values were omitted from percentage calculation

*CCI* Charlson Comorbidity Index, *HADS* Hospital Anxiety and Depression Scale

**Table 5:** Factors associated with the use of nonprescription medications by logistic regression analysis (*n* = 623)

Variables	Univariate	Multivariate	*P* value
	Crude OR (95% CI)	Adjusted OR (95% CI)	
Female sex	1.47 (1.08–2.02)	1.58 (1.03–2.41)	0.036
Age≧75	0.72 (0.52–0.98)	0.78 (0.55–1.11)	0.171
Education > high school	1.68 (1.21–2.34)	1.70 (1.16–2.49)	0.007
Economic status (ref. = average)			
Less than average	1.09 (0.70–1.70)	1.20 (0.72–1.99)	0.484
More than average	1.89 (1.30–2.77)	1.62 (1.07–2.43)	0.021
Never smoker	1.48 (1.08–2.03)	1.23 (0.81–1.88)	0.330
Anxiety by HADS	1.17 (0.76–1.80)	1.27 (0.77–2.10)	0.350
Depression by HADS	1.05 (0.72–1.54)	1.23 (0.79–1.91)	0.367

*HADS*0 Hospital Anxiety and Depression Scale

Subgroup analyses were conducted each for dietary supplements and OTC drugs. In logistic regression analysis which assessed factors related to the use of dietary supplements, it was shown that female sex (Adjusted OR = 1.61, 95% CI = 1.03–2.51) and higher educational attainment (Adjusted OR = 1.73, 95% CI = 1.16–2.58) were significantly associated the use of dietary supplements, though lower economic status did not reach at a significant level (Adjusted OR = 1.47, 95% CI = 0.96–2.24). In logistic regression analysis which assessed factors related to OTC use, it was shown that only female sex (Adjusted OR = 1.61, 95% CI = 1.03–2.51) was associated with the OTC use. Although the other variables showed no significant association with OTC use because of the small number of subjects (*n* = 59), there was a similar tendency in dietary supplements users and OTC users [see Additional file 1].

### Polypharmacy and nonprescription medications

Patients who were prescribed more than 5 drugs accounted for 39.5% of the participants. Of all participants, 89 (12.2%) patients were concurrently using nonprescription medications with more than 5 prescription medications. Polypharmacy and the use of nonprescription medication were not significantly associated significantly on univariate analysis.

## Discussion

The use of dietary supplements and OTC drugs was common in elderly patients with chronic diseases, and its use is associated with female sex, higher education, and good economic status. Concurrent use of nonprescription medications with more than 5 prescription medications was not uncommon, but the disclosure rate to the physician was low.

This study showed that 32.5% of Japanese elderly patients with prescription medications for chronic diseases concurrently used nonprescription medication. Previous studies have reported that the use of nonprescription medications is 16.6–66% in the elderly population [15–18], and 20.2–71.4% in the general population [19–21]. The percentage of patients using nonprescription medication in the present study is consistent with previous reports. However, the percentage in our study may be higher than in the general elderly population as all the participants in the present study have chronic diseases with prescription medications.

The present study also demonstrated that dietary supplements are the most commonly used nonprescription medications. Among dietary supplements, vitamins and minerals were the most frequently used, followed by *aojiru* (green juice) and chondroitin-glucosamine. One study conducted in a hospital in Tokyo reported that health foods including dietary supplements and herbal medicine were commonly used as complementary and alternative medicine (CAM) [22]. Compared to a U.S. report, non-vitamin dietary supplements were different in our study. Echinacea, Ginseng, *Ginkgo biloba*, and St John’s wort were reported to be popular in the U.S. [7], but such products were not commonly used in the present study. Vitamins and minerals are universally common supplements but other supplements can vary per country and region, thereby reflecting the differences in health system, OTC medications approval, and culture. *Aojiru* (Green juice), a juice of mixed green vegetables, is very popular in Japan. It is usually not harmful for healthy adults, but it can be risky for patients with chronic kidney disease, as it is enriched with potassium. Chondroitin-glucosamine is one of the most popular supplements in the world, as many elderly patients have orthopedic problems. In this study, use of OTC medications and herbal medicine was less frequent than previously reported in Japan. The reason for this may be that the clinic provides mainly conventional medicines and the patients who prefer to use OTC medications or herbal medicines do not come to the clinic.

Predictors for the use of nonprescription medications were consistent with previous studies. Female sex [2, 7, 15, 19, 21, 23], younger age [2, 16], higher educational qualifications [7, 15, 23], lower income [24], higher income [16], absence of smoking habits [15], living alone [15, 18], retirement [15], and chronic conditions [7] have been reported to be associated with the use of dietary supplements or OTC medications. Contrary to our hypothesis, anxiety, depression, and polypharmacy were not associated with the use of nonprescription medications.

Concurrent use of nonprescription medications with more than 5 prescription medications was frequent in as much as 12.2% of participants. Previous studies also demonstrated high numbers of patients on concurrent use of nonprescription and prescription medications [3, 25, 26]. Elderly patients with chronic diseases are at high risk of drug-drug interaction due to the concurrent use of medications. Research from the U.S. reported the potential interaction between some nonprescription and prescription medications, especially with anticoagulant and antiplatelet therapies [3, 27]. However, one study reported that the actual potential for harm by interaction between prescription medications and dietary supplements was low [28]. In Japan, the risks of interaction between nonprescription medications and prescription medications may be different from other countries because St John’s wort, ginseng, ginkgo, or garlic, which can interact with anticoagulant and antiplatelet therapies, were rarely used in this study.

The percentage of patients given disclosure about their use of nonprescription medications to the physician was low. According to previous studies, 33% to 48.6% of patients taking dietary supplements disclosed this to a healthcare provider [15, 29]. Non-disclosure rates of CAM including dietary supplements were 29% to 77% according to one review [30], recent studies also supported the previous results and questions about the use by a medical practitioner was a major predictor of disclosure [31–33]. It is important to collect all information about nonprescription medications considering the potential risk of drug-drug interactions. Medical practitioners should recognize the potential use of dietary supplements and try to ask patients whether they use nonprescription medications. Education for both medical practitioners and patients would be important to increase the disclosure rate. In addition, patients that use dietary supplements should know the potential harm and need to ask doctors if they can take the product, especially elderly patients with chronic diseases who use prescribed drugs.

There are some limitations in the present study. First, this study was conducted at a single facility, so selection bias could have happened. The use of nonprescription medications may be higher in Tokyo than other areas, because the average income in Tokyo is higher than in other areas in Japan. Secondly, there is a possibility of underreporting the use of nonprescription medications, as this study was conducted using a self-administered questionnaire. The rate of reporting the use of supplements through a self-administered questionnaire was demonstrated to be very low [34], but previous studies were also mainly conducted by questionnaires and our data is therefore comparable with others.

As a result, we reported the use and details of nonprescription medications in elderly patients with chronic diseases and its predictors, which are relatively new findings for Asian countries. Further research is needed to examine the relationship between the use of nonprescription medications and actual harm by interactions caused by concurrent use of nonprescription medications and prescription medications.

## Conclusions

Our results show that the use of nonprescription medications was common in elderly patients with chronic diseases. The type of the nonprescription medications were mainly dietary supplements including vitamins/minerals, *aojiru* (green juice), and chondroitin-glucosamine. Female sex, higher educational qualifications, and good economic status were associated with the use of nonprescription medications. Concurrent use of nonprescription and prescription medications, even with more than 5 prescription medications, were common in elderly patients, and the disclosure rate of the use of nonprescription medications was low. Therefore, medical practitioners should ask about the use of nonprescription medications and assess the potential drug-drug interaction with prescription medications.

## Additional file

Additional file 1: Factors associated with the use of dietary supplements and OTC drugs (subgroup analyses). Logistic regression analyses were conducted for assessing factors associated with the use of dietary supplements and OTC drugs. (DOCX 23 kb)

## Abbreviations

CAM: Complementary and alternative medicine
CCI: Charlson Comorbidity Index
CI: Confidence interval
GP: General practitioner
HADS: Hospital Anxiety and Depression Scale
IQR: Interquartile range
OR: Odds ratio
OTC: Over-the-counter
SD: Standard deviation
U.S.: The United States
