# Supplementary material for: Factors associated with the use of dietary supplements and over-the-counter medications in Japanese elderly patients
Source: BMC Fam Pract. 2018 Jan 24;19:20. doi: 10.1186/s12875-017-0699-9 (PMC5784694; doi:10.1186/s12875-017-0699-9)
Supplement: Additional file 1: — Factors associated with the use of dietary supplements and OTC drugs (subgroup analyses). Logistic regression analyses were conducted for assessing factors associated with the use of dietary supplements and OTC drugs. (DOCX 23 kb) [file 12875_2017_699_MOESM1_ESM.docx]

Additional file 1. Factors associated with the use of dietary supplements and OTC drugs (subgroup analyses)

| **Variables** | Dietary supplements (n=181) | | OTC drugs (n=59) | | |
| --- | --- | --- | --- | --- | --- |
|  | **Adjusted OR**  **(95% CI)** | *P* value | | **Adjusted OR**  **(95% CI)** | *P* value |
| Sex: Female | 1.61 (1.03-2.51) | 0.035 | | 2.76 (1.35-5.62) | 0.005 |
| Age≧75 | 0.86 (0.59-1.25) | 0.429 | | 1.04 (0.59-1.83) | 0.902 |
| Educational qualification>high school | 1.73 (1.16-2.58) | 0.007 | | 1.12 (0.62-2.04) | 0.705 |
| Economic status (ref.=average) |  |  | |  |  |
| Less than average | 1.01 (0.59-1.74) | 0.963 | | 1.03 (0.46-2.33) | 0.944 |
| More than average | 1.47 (0.96-2.24) | 0.074 | | 1.46 (0.77-2.76) | 0.245 |
| Smoking status: Never smoker | 1.31 (0.85-2.04) | 0.222 | | 0.72 (0.37-1.43) | 0.351 |
| Anxiety by HADS | 1.02 (0.60-1.73) | 0.956 | | 1.74 (0.82-3.68) | 0.150 |
| Depression by HADS | 1.03 (0.64-1.64) | 0.917 | | 0.61 (0.28-1.33) | 0.212 |

Missing values were omitted from percentage calculation

OTC: over-the-counter, HADS: Hospital Anxiety and Depression Scale
